# The Utility of Routine Ultrasound Imaging after Elective Transplant Ureteric Stent Removal

**DOI:** 10.1155/2016/1231567

**Published:** 2016-07-14

**Authors:** Bibek Das, Dorian Hobday, Jonathon Olsburgh, Chris Callaghan

**Affiliations:** Department of Nephrology and Transplantation, Guy's Hospital, Great Maze Pond, London SE1 9RT, UK

## Abstract

*Background*. Ureteric stent insertion during kidney transplantation reduces the incidence of major urological complications (MUCs). We evaluated whether routine poststent removal graft ultrasonography (PSRGU) was useful in detecting MUCs before they became clinically or biochemically apparent.* Methods*. A retrospective analysis was undertaken of clinical outcomes following elective stent removals from adult single renal transplant recipients (sRTRs) at our centre between 1 January 2011 and 31 December 2013.* Results*. Elective stent removal was performed for 338 sRTRs. Of these patients, 222 had routine PSRGU (median (IQR) days after stent removal = 18 (11–31)), 79 had urgent PSRGU due to clinical or biochemical indications, 12 had CT imaging, and 25 had no further renal imaging. Of the 222 sRTRs who underwent routine PSRGU, 210 (94.6%) had no change of management, three (1.4%) required repeat imaging only, and eight patients (3.6%) had incidental (nonureteric) findings. One patient (0.5%) had nephrostomy insertion as a result of routine PSRGU findings, but no ureteric stenosis was identified. Of 79 patients having urgent PSRGU after elective stent removal, three patients required transplant ureteric reimplantation.* Conclusions*. This analysis found no evidence that routine PSRGU at two to three weeks after elective stent removal provides any added value beyond standard clinical and biochemical monitoring.

## 1. Introduction

Major urological complications (MUCs) after kidney transplantation occur in less than 10% of recipients but are associated with significant morbidity and occasional mortality [1]. These complications are related to the transplant ureter and vesicoureteric anastomosis and present as either urinary leaks or collecting system obstruction, usually within the first 3 months after transplantation [1]. Urinary leaks can lead to enlarging deep collections and/or leakage of urine through the wound. Obstruction due to ureteric stenosis may present with oliguria or, rarely, a swollen graft. Both MUCs usually lead to elevated serum creatinine and can be detected on an ultrasound scan (USS).

Many transplant centres insert a transplant ureteric stent at the time of kidney transplantation in order to protect the vesicoureteric anastomosis. While the main disadvantage with ureteric stent placement is an increased risk of urinary tract infections [2, 3], recent Cochrane reviews concluded that universal prophylactic ureteric stenting is effective at reducing the incidence of MUCs [4, 5].

The optimal time of removal of the transplant ureteric stent is currently unknown, though many units remove the stent electively at 4–6 weeks after transplant. Removal of the stent can reveal underlying MUCs that have hitherto been masked by the presence of the stent. For this reason, our unit has routinely performed poststent removal graft ultrasonography (PSRGU). This is performed in an attempt to identify features of MUCs, such as hydronephrosis or deep urine collections, before they become clinically or biochemically apparent.

Anecdotally, there is variation between units as to if (or when) a PSRGU is performed. We are not aware of any previous studies or guidelines that address whether routine PSRGU is beneficial and what the optimal timing is, if it is of value. Given that posttransplant stent removal occurs during a period of intense outpatient clinical and biochemical monitoring, it is possible that routine PSRGU does not aid in the recognition of MUCs, as clinical and/or biochemical abnormalities may lead to urgent imaging investigations before a routine PSRGU is performed.

Therefore, we evaluated whether our practice of routine ultrasound imaging after elective transplant ureteric stent removal changed management, beyond regular clinical review and assessment of renal function. In particular, we wished to identify any patients who required ureteric interventions after initial detection on a routine PSRGU.

## 2. Methods

This was a retrospective analysis of all adult renal transplants (including simultaneous pancreas-kidney (SPK) transplants) performed from 1 January 2011 to 31 December 2013 and who had their stent removed, at our unit. Follow-up ceased on 1 March 2014. Data were retrieved from electronic case notes and pathology databases.

All kidney transplants performed in our unit have a double J transplant ureteric stent inserted (6 Fr or 7 Fr). All surgeons use the extravesical Lich-Gregoir ureteroneocystostomy technique, with either interrupted or continuous 4/0 polydioxanone sutures, according to preference. Immunosuppression consists of basiliximab, tacrolimus, mycophenolate mofetil, and prednisolone. A small number of patients (<10%) received alemtuzumab at induction, rather than basiliximab, either as part of a clinical trial [6] or if they had historic or low level donor-specific anti-HLA antibodies with a negative flow cytometric cross-match.

The frequency of outpatient follow-up appointments in the first year after renal or SPK transplantation is summarised in Table 4. Ureteric stents are removed using flexible cystoscopy at 4–6 weeks after transplantation (“elective stent removal”), unless there is a clinical indication to remove them earlier (“urgent stent removal”). A routine USS was requested at the first clinic visit after stent removal (“routine USS”). At every clinic appointment, a doctor or transplant nurse specialist checked for symptoms consistent with MUCs and the recipient's serum creatinine was measured.

A sustained increase in serum creatinine more than 15% from baseline in the period after stent removal, or other symptoms or signs of possible graft dysfunction, led to urgent graft imaging, usually by USS (“urgent USS”). An elevated serum creatinine with a dilated renal pelvis (>12–15 mm diameter) led to insertion of a percutaneous nephrostomy and nephrostogram. Ureteric stenoses were preferentially managed by surgical revision.

For this analysis, data collected included donor and recipient variables (age, donor type, and transplant type) and operative data (number of ureters and stents). The date of the stent removal was recorded, including whether this was an elective or urgent procedure (i.e., removed earlier than planned). The date and full report of any relevant renal transplant imaging after stent removal was recorded, and the subsequent management was clarified by cross-checking electronic case notes and outpatient clinic letters. Relevant renal imaging included imaging modalities suitable for detecting hydronephrosis or urinary leaks, such as ultrasonography, CT, MRI, nephrostogram, and nuclear medicine imaging (e.g., MAG-3). Any ureteric surgery or interventions performed until the end of the follow-up period were recorded, regardless of PSRGU findings.

Recipients were excluded from the analysis for the following: (1) if the stent removal was performed at another hospital; (2) if they received a double kidney transplant (adult or paediatric donor), as outflow obstruction of one of the grafts may not have produced a significant deterioration in renal function if the other graft in the pair was unobstructed and functioning well; (3) if transplant nephrectomy occurred prior to elective stent removal; (4) those who had early stent removal as part of a clinical trial (Transplant Ureteric Stent Trial, ISRCTN 09184595).

Adult single renal transplant recipients (sRTRs) who underwent routine USS after elective stent removal were grouped into one of four categories, based on their clinical management following their PSRGU: (1) patients who had no change in their management; (2) patients who underwent more intensive monitoring or further investigations but did not undergo any ureteric intervention (e.g., more frequent monitoring of renal function, a repeat ultrasound scan, or MAG-3 investigation); (3) patients who required invasive intervention for possible MUCs (defined as nephrostomy, urethral catheter reinsertion for MUC, stent reinsertion, or ureteric surgery); (4) patients who required further investigation or management for incidental findings (e.g., transplant renal artery stenosis, collections, or parenchymal lesions).

## 3. Results

### 3.1. Patient Groups

**Figure 1:**
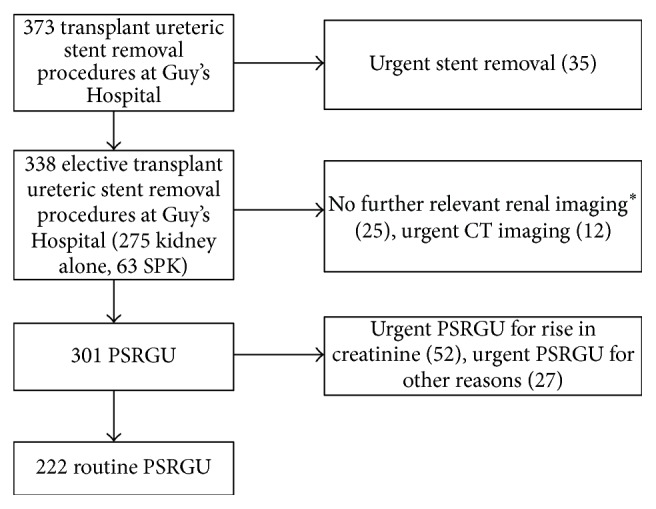
Flow chart summarising recipients of single kidney transplants undergoing stent removal at Guy's Hospital. ^*∗*^Seven patients were followed up in units where routine PSRGU are not performed.

During the study period, 373 patients were identified who met our inclusion criteria. The study pathway is summarised in Figure 1. Of those patients, 35 had their stents removed urgently (median (interquartile range, IQR) number of days after transplant = 21 (14–31.5)). Four of these patients subsequently underwent ureteric reimplantation for ureteric stenosis (4/35; 11.4%). All of these patients had graft hydronephrosis detected on urgent USS, not routine PSRGU. The indications for urgent stent removal are summarised in Table 1.

This left 338 sRTRs (221 males) who underwent elective transplant ureteric stent removal at our centre. Mean (standard deviation) donor age was 46.0 (15.4) years, with mean (standard deviation) recipient age of 45.2 (13.3) years. One hundred and thirty eight kidneys came from live donors, with 112 from donation after brain death donors and 88 from Maastricht category III donation after circulatory death donors. Stents were removed at a median (IQR) of 44 (40–49) days after transplant.

Thirty-seven patients did not have a PSRGU within 6 months; 25 had no relevant renal imaging and 12 patients had urgent CT imaging. None of these patients required any form of ureteric intervention until the end of the follow-up period.

Therefore, PSRGU was performed in 301 patients after elective stent removal at our hospital. A routine PSRGU was performed on 222 patients at a median (IQR) of 18 (11–31) days after stent removal. Urgent PSRGU was required for 79 patients at a median (IQR) of 6 (4–14) days after stent removal. The detailed indications for urgent USS after elective stent removal are shown in Table 2. The majority (66%) were performed for a rise in serum creatinine.

### 3.2. Outcomes of USS after Elective Stent Removal

Clinical outcomes following routine and urgent USS after elective stent removal are summarised in Table 3. After routine PSRGU the overwhelming majority of patients had no change to management (*n* = 210, 94.6%). Three patients (1.4%) with mild hydronephrosis but stable renal function required repeat imaging only. Eight patients (3.6%) had further imaging or interventions due to incidental findings on routine PSRGU: four recipients with small perinephric collections (no intervention needed); one asymptomatic large residual bladder volume caused by benign prostatic hyperplasia (eventually requiring transurethral resection of the prostate); and one possible transplant renal artery stenosis not confirmed on subsequent ultrasonic angiography. In addition, there was one case of a small collection causing hydronephrosis (anteroposterior diameter at renal pelvis = 22 mm) in the context of stable renal function and no signs of sepsis; the patient underwent an elective CT-guided drainage of this collection that was complicated by systemic sepsis. The patient was treated with broad-spectrum antibiotics and later discharged. There was no lasting effect on renal function. The last incidental finding was a possible arteriovenous fistula. This was excluded by means of a duplex ultrasound, which instead showed possible TRAS. This was in the context of stable renal function and mild hypertension on a single agent with no prior suspicion of TRAS. The patient underwent an angioplasty which was complicated by capsular perforation and infarction requiring transplant nephrectomy.

One patient (0.5%) required a nephrostomy based on a strong suspicion of ureteric obstruction after a routine PSRGU. The ureteric stent was removed earlier than usual at day 23 after operation to facilitate discharge back to their transplant centre overseas. The routine poststent removal ultrasound scan was performed at day 30 which showed mild hydronephrosis with a renal AP diameter 12 mm and no collections. A repeat ultrasound scan performed at day 34 showed worsening hydronephrosis (renal anteroposterior diameter 22 mm), and the serum creatinine was rising; therefore a percutaneous nephrostomy was performed. The subsequent nephrostogram, however, showed prompt drainage of contrast into the bladder with no evidence of obstruction, and the nephrostomy was removed. A biopsy at day 37 showed evidence of acute cellular rejection that received medical management. The patient did not require any further surgical or interventional treatment for suspected MUC until the end of the follow-up period.

**Figure 2:**
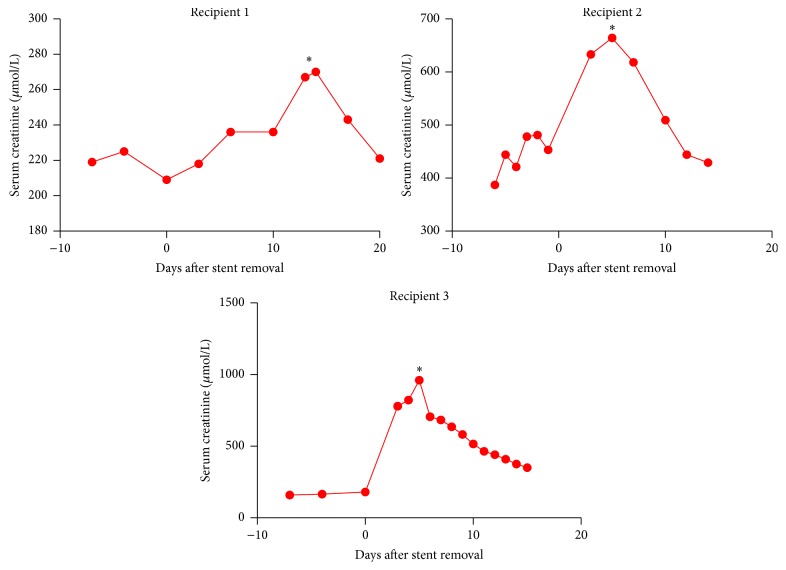
Trends in serum creatinine after elective stent removal for three recipients who required ureteric reimplantation for ureteric stenosis. Day 0 corresponds to the date of ureteric stent removal. Date of urgent PSRGU and nephrostomy insertion is highlighted by an asterisk (*∗*).

Importantly, four patients required ureteric intervention after urgent PSRGU for elevated creatinine; all had a nephrostomy with three eventually requiring ureteric reimplantation for ureteric stenosis while one patient had no obstruction on nephrostogram. The trend in serum creatinine after stent removal for the three patients with ureteric stenosis is displayed in Figure 2. No urinary leaks were identified on any PSRGU, though one urine leak occurred at two weeks after transplantation with a stent in situ. The overall MUC rate in this group was 1.2% (4/338).

## 4. Discussion

Ureteric stent insertion at the time of kidney transplantation is widely practised and is performed with the aim of reducing the incidence of MUCs. MUCs are rare but often become apparent soon after stent removal. This study aimed to determine whether routine ultrasound imaging of transplant kidneys two weeks after elective ureteric stent removal can detect MUCs before they become clinically apparent. To our knowledge there are no other studies or national guidelines that address this issue.

The main finding from this study was that the overwhelming majority of patients who underwent a routine USS at approximately two weeks after elective stent removal did not require an intervention or change of management as a result of the USS. No MUCs were identified in the 222 patients who underwent a routine PSRGU. Instead, ureteric stenoses causing hydronephrosis were detected soon after stent removal on urgent PSRGU, prompted by rises in serum creatinine within 14 days of stent removal.

One study has suggested that it could be useful to carry out serial screening ultrasounds at two weekly intervals to detect postoperative complications such as ureteric obstruction and perinephric fluid collections [7]. This paper, however, does not mention the use of ureteric stents and our data suggests that this practice is unlikely to be beneficial due to the low rate of significant pathology detected on PSRGU in the absence of graft dysfunction. It could be hypothesised that routine PSRGUs performed earlier after elective stent removal may be beneficial in detecting mild hydronephrosis before graft function deteriorates. However, we note that, of the three patients who required ureteric reimplantation, two had creatinine rises within five days of stent removal. This suggests that there is a very narrow window within which routine PSRGU may be advantageous. Given that elective transplant ureteric stent removal occurs during a period of intense clinical and biochemical monitoring in outpatients, it is difficult to envisage how routine PSRGU would be beneficial beyond frequent measurement of graft function.

Another finding was that the detection of incidental lesions on routine PSRGU could lead to unnecessary, or even harmful, investigations or interventions. The potential harm from identifying incidental findings has been discussed widely in the literature in the context of image-based screening [8–10]. It has also been highlighted in recent campaigns that overtreatment of findings that would never have become clinically relevant may result in both physical and psychological harm, as well as incurring additional costs [11, 12]. Financial implications of performing unnecessary investigations are particularly relevant in the current financial climate. In this study, intervention in a patient with presumed transplant renal artery stenosis in the context of stable renal function and blood pressure incurred the loss of a functioning graft. Also, an attempt to drain a small collection that was not infected or compromising renal function led to a patient developing sepsis. Both cases highlight the danger in treating incidental findings that are not having any demonstrable effect on the patient's clinical condition or graft function. There was possibly one patient where routine PSRGU identified a finding that warranted intervention. This patient had an asymptomatic large residual bladder volume secondary to benign prostatic hyperplasia. Prior to this being discovered on PSRGU, his renal function was stable and he had not had any urinary tract infections.

This study identified that the group of patients having urgent stent removal appeared to have an increased rate of MUCs (11.4%). This was a ten-fold increase on the rate of MUC in those undergoing elective stent removal (3/338; 0.9%). As detailed in Table 1, these patients had early urological complications, predominantly urinary tract infections. The aetiology of the high rate of transplant ureteric stenosis in this group is unclear, but this group may warrant closer monitoring, including scheduled early USS after their urgent stent removal.

Our unit policy has changed based on the findings of this study, with specific criteria for requesting a routine USS after elective stent removal:

1. Recipients where there is more than one transplant ureter (e.g., double kidney transplants, paediatric en bloc double kidney transplants, and multiple ureters from a single kidney).
2. Recipients who are still dialysis-dependent at the time of stent removal.
3. Recipients where the transplant ureter has been reconstructed or has undergone other complex ureteric surgery.

These criteria reflect the difficulty in relying on serum creatinine measurement to detect MUCs in patients with multiple ureters or high baseline creatinine.

The main limitation of this study is the possibility that it is underpowered due to a low MUC rate after elective stent removal (0.9%). The overall MUC rate in our study population was 2.1% (8/373); this is similar to other recent studies [13–15]. In addition, routine PSRGU were done at a median of 18 days after elective stent removal. If these PSRGU had been done earlier (e.g., 3–5 days), it is possible that hydronephrosis may have been detected prior to a dramatic serum creatinine rise, with the opportunity for earlier urinary tract decompression, and the possible avoidance of severe graft dysfunction. This is demonstrated by the clinical time course seen in recipients 2 and 3 (Figure 2).

In conclusion, this study evaluating routine ultrasonography after elective transplant ureteric stent removal failed to show any apparent benefit compared to regular clinical and biochemical review. There remain unanswered questions on the optimal monitoring of specific patient groups, such as the paediatric population, double kidney transplant recipients, duplex kidneys, patients undergoing urgent stent removal, and those candidates suitable for early stent removal. With a current trend toward earlier ureteric stent removal in order to minimise stent-related complications [16], there will likely be further interest in determining how to best monitor for MUCs in this group of patients. Overall, we emphasise the need for close clinical and biochemical monitoring in the postoperative period and a need to standardise practise for all transplant recipients.

## Figures and Tables

**Table 1:** Indications for urgent transplant ureteric stent removal.

Indication	*n*
Urinary tract infection	22
Infected perinephric collection	3
Stent migration	3
Suprapubic pain	3
Urinary incontinence	2
Hydronephrosis with stent in situ	1
Clot retention after graft biopsy	1

*Total*	*35*

**Table 2:** Indications for urgent USS after elective stent removal.

Indication	*n*
Serum creatinine rise	52
Pain over the graft	9
Oliguria	4
Sepsis	3
Peripheral oedema	2
Pus from wound	2
Swelling over the graft	2
To assess the size of recently drained lymphocele	1
Previous ureteric injury	1
Previous ureteric reimplantation for postoperative urinary leak	1
Fluid leaking from the incision	1
Suspected blocked drain	1

*Total*	*79*

**Table 3:** Clinical outcomes following routine and urgent USS after elective stent removal.

Outcome	Routine PSRGU (*n* = 222)	Urgent PSRGU for rise in serum creatinine (*n* = 52)	Urgent PSRGU for reason other than rise in serum creatinine (*n* = 27)
No change in management (%)	210 (94.6)	42 (80.8)	19 (70.4)
Repeat imaging or more intensive monitoring, but no ureteric intervention (%)	3 (1.4)	5 (9.6)	1 (3.7)
Invasive intervention for suspected MUC^*∗*^ (%)	1 (0.5)	4 (7.7)	0 (0)
Further investigation or intervention for incidental findings (%)	8 (3.6)	1 (1.9)	7 (25.9)

^*∗*^Defined as nephrostomy, urethral catheter reinsertion for MUC, stent reinsertion, or ureteric surgery.

**Table 4:** Schedule of follow-up outpatient clinic appointments in the first year after kidney or SPK transplantation at Guy's hospital.

Month	Week 1					Week 2					Week 3					Week 4				
	M	T	W	T	F	M	T	W	T	F	M	T	W	T	F	M	T	W	T	F
1	Inpatient					o		•		o	o		•		o	o				•
2	o			•		o			•		o			•			•			
3			•						•				•						•	
4			•						•					•					•	
5			•											•						
6			•											•						
7									•											
8									•											
9									•											
10									•											
11									•											
12									•											

o: nurse's clinic; •: doctor's clinic.
